# Unusual computed tomography findings of radionecrosis after chemoradiation of stage IV hypopharyngeal cancer: a case report

**DOI:** 10.1186/1752-1947-5-25

**Published:** 2011-01-20

**Authors:** Yuh Baba, Yasumasa Kato, Kaoru Ogawa

**Affiliations:** 1 Department of Otorhinolaryngology, Ohtawara Red Cross Hospital, 2-7-3 Sumiyoshi-cho, Ohtawara City, Tochigi 324-8686, Japan; 2 Department of Otorhinolaryngology, Head and Neck Surgery, Keio University, 35 Shinanomachi Shinjuku, Tokyo 160-0082, Japan; 3 Department of Biochemistry & Molecular Biology, Kanagawa Dental College, Yokosuka 238-8580, Japan

## Abstract

**Introduction:**

Radionecrosis (post-radiotherapy laryngeal submucosal inflammation and necrosis) is a complication of (chemo) radiotherapy for hypopharyngeal cancer that is difficult to differentiate from tumor recurrence.

**Case presentation:**

A 67-year-old Japanese man presented with a condition extremely difficult to diagnose differentially as radionecrosis or tumor recurrence after radiotherapy for hypopharyngeal cancer. Although tumor recurrence was suspected from clinical conditions and computed tomography findings, pathologic analysis revealed no evidence of tumor recurrence, and successful therapy with steroids and antibiotics reduced the mucosal edema.

**Conclusion:**

Our findings emphasize the wide spectrum of radiographic presentation of radionecrosis after chemoradiation of stage IV hypopharyngeal cancer.

## Introduction

Adverse reactions to radiotherapy can include both early and late complications. Late complications may arise more than several months after the completion of radiotherapy, even in patients who show no evidence of complications during exposure. These complications are generally irreversible, progress gradually, and cause crucial organ dysfunction. Late radiotherapy complications involving the head and neck area include disturbances of salivary secretion, jaw necrosis, chondronecrosis, hypothyroidism, subcutaneous tissue fibrosis, and development of second cancers. Improvements in radiotherapy seemed to have reduced the incidence of radiotherapy-related adverse reactions, from 5% during the 1970s to 1% during the 1990s [1,2]. Concomitant chemoradiation has become widely used in patients with advanced hypopharyngeal cancer, with an incidence of adverse reactions that has been predicted to increase in the future. To the best of our knowledge, however, few reports are available about computed tomography (CT) findings in patients with this condition [3,4]. We describe here a patient who experienced radionecrosis (post-radiotherapy laryngeal submucosal inflammation and necrosis) after chemoradiation of stage IV hypopharyngeal cancer, focusing on CT findings.

## Case presentation

A 67-year-old Japanese man visited our department complaining of pharyngeal pain. He had no appreciable medical or family histories, had smoked 30 cigarettes per day for 35 years, and had no drinking history. The patient had become aware of pharyngeal pain and had visited his general practitioner, but his condition did not improve.

**Figure 1:**
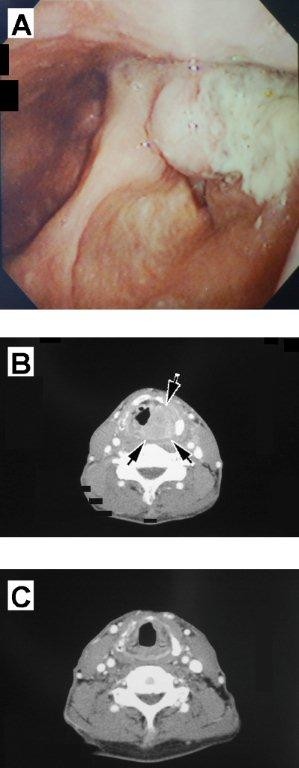
**Before and after chemoradiotherapy**. **(a) **Fiberoptic endoscopy showing a neoplastic lesion in the left pyriform recess. **(b) **CT showing a tumor occupying the left pharyngeal cavity and infiltrating the thyroid cartilage. **(c) **CT performed after the completion of chemoradiotherapy, showing no evidence of neoplastic lesions.

At his initial visit to our department, laryngopharyngeal fiberoptic endoscopy revealed a neoplastic lesion in the left pyriform recess (Figure 1a). Vocal cord fixation was also observed. Pathologic examination of a biopsy specimen revealed a poorly differentiated squamous cell carcinoma (SCC), and contrast CT showed a tumor occupying the left pharyngeal cavity and infiltrating the thyroid cartilage (Figure 1b). Lymph node metastasis in the left side of the neck was confirmed, but no distant metastases were found. The patient was diagnosed with stage IVA hypopharyngeal cancer (T4aN2bM0) and received two cycles of docetaxel, cisplatin, and 5-fluorouracil as neo-adjuvant chemotherapy. Three weeks after neo-adjuvant chemotherapy, he was started on concomitant chemoradiation, consisting of 10 mg/m^2 ^docetaxel on days 1, 8, 15, 22, and 29, and 3D radiotherapy, consisting of 2Gy fractions once daily for five days per week, for a total dose of 62.0Gy in 31 fractions. The dose to the whole neck was 40.0Gy, and the dose to the primary site was 62.0Gy. Although the patient complained of mild pain in the throat, he was able to eat a sufficient number of light meals.

At approximately one month after completing radiotherapy, we took a biopsy sample of his left hypopharynx under direct laryngoscopy to evaluate response to therapy, as biopsy is done after initial treatment in the UMCC (University of Michigan Comprehensive Cancer Center) protocol 9520 [5]. Pathologic examination showed no obvious presence of remaining tumor, and CT showed no obvious neoplastic lesions (Figure 1c). The patient recovered his pretreatment vocal cord fixation and achieved a pathologic complete response (CR).

**Figure 2:**
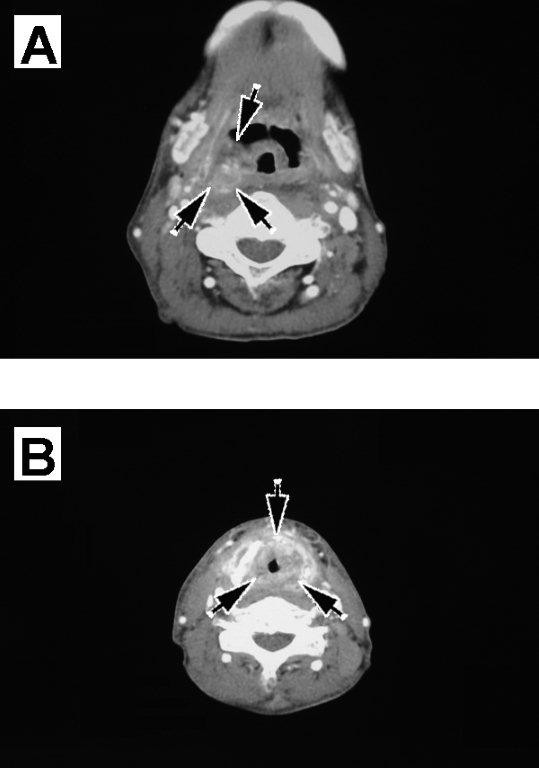
**Follow-up CT results**. **(a) **A suggestive space-occupying lesion in the right lateral pharyngeal wall at the level of the epiglottis, and **(b) **a suggestive circumferential space-occupying lesion in the mucosa and airway constriction at the level of the thyroid cartilage.

**Figure 3:**
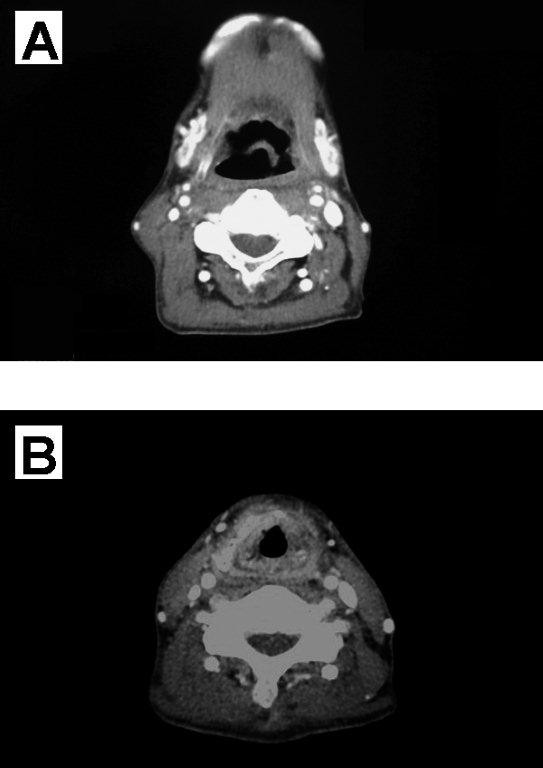
**CT results after administration of steroids and antibiotics**. These show the disappearance of space-occupying lesions initially suspected as tumor recurrence at the levels of the **(a) **epiglottis and **(b) **thyroid cartilage.

Later, as an outpatient, he gradually developed pharyngeal pain and dyspnea. Laryngopharyngeal fiberoptic endoscopy showed mucosal swelling in the pharynx and larynx. CT images of his neck, taken approximately eight months after the completion of radiotherapy, revealed a suggestive space-occupying lesion in the right lateral pharyngeal wall at the level of the epiglottis and a suggestive circumferential space-occupying lesion in the mucosa and airway constriction at the level of the thyroid cartilage. In detail, CT scans showed mixed masses of enhanced and non-enhanced lesions at both the epiglottis and thyroid-cartilage levels, strongly indicating tumor recurrence. Although we observed swelling of the surrounding soft tissues and segmentation or collapse of the thyroid cartilage, we did not observe defluxion of the arytenoid cartilage or abnormal gases contacting the cartilage (Figure 2a,b). Dyspnea was gradually aggravated, and the patient underwent a tracheostomy. Under general anesthesia, we removed biopsy samples from his lateral pharyngeal wall and larynx. Pathologic examination of these biopsy samples revealed no remaining cancer. Because the significant mucosal edema suggested the possibility of undetected submucosal recurrent lesions, we took additional biopsy samples under general anesthesia, but pathologic examination again showed no evidence of remaining cancer, although significant tissue degeneration and necrosis were observed. After a tentative diagnosis of radionecrosis after radiotherapy, the patient was treated with steroids and antibiotics. His laryngeal edema and swelling in the right lateral pharyngeal wall improved gradually, resulting in significant reductions in pharyngeal pain and pain during swallowing, allowing the patient to ingest solid foods. Moreover, the space-occupying lesion, which had been suspected as being tumor recurrence, had disappeared (Figure 3a,b). After CT evaluation, the tracheostomy was successfully reversed.

Two years after primary treatment with radiation, no evidence has been found of tumor recurrence. The patient's speech and deglutition were preserved, and his voice quality was within normal limits.

## Discussion

Radionecrosis is one of the late complications of radiotherapy. Although its incidence is approximately 1% [1,2], it is predicted to increase because of the increasing use of concomitant chemoradiotherapy. One possible mechanism of pathogenesis is vasculitis-induced narrowing and occlusion of the intravascular lumens [6]. Although radionecrosis usually occurs within the first year after radiotherapy [7], it may occur more than ten years later [8]. Treatment regimens for radionecrosis include application of antibiotics and steroids, hyperbaric oxygen therapy, and surgical intervention [9].

The clinical manifestations of radionecrosis, including hoarseness, difficulties with oral intake, pharyngeal pain, dyspnea, and skin fistula, are similar to those of tumor recurrence [10]. In addition, although radionecrosis is frequently accompanied by various CT findings, including swelling of the surrounding soft tissues, segmentation or collapse of the thyroid cartilage, defluxion of the arytenoid cartilage, and abnormal gases in contact with the cartilage, all of these findings are regarded as nonspecific [3,4]. Moreover, only about 50% of patients with radionecrosis present with these CT findings [11]. CT images of the neck of our patient revealed a suggestive space-occupying lesion in the right lateral pharyngeal wall at the level of the epiglottis and a suggestive circumferential space-occupying lesion in the mucosa and airway constriction at the level of the thyroid cartilage. In detail, CT scans showed mixed masses of enhanced and non-enhanced lesions at both the epiglottis and thyroid cartilage levels, masses that could have been easily mistaken for tumor recurrence. The enhanced lesions were likely viable infectious cells, consistent with the effectiveness of the antibiotics, whereas the non-enhanced lesions were likely necrotic cells.

In addition to the similarity of clinical conditions and CT findings in patients with radionecrosis and recurrent lesions, some submucosal recurrent lesions may not be detected by biopsy under direct laryngoscopy, especially in patients with significant mucosal edema. Moreover, in some patients, radionecrosis and recurrent tumor may occur concurrently. Thus, differential diagnosis between radionecrosis and tumor recurrence is considered extremely difficult. Positron emission tomography- (PET)-CT is an alternative imaging modality that has been successfully used to differentiate postradiation changes, radionecrosis, and tumor recurrence. Furthermore, Fludeoxyglucose- (FDG)-PET scanning after radiation can predict tumor regrowth three months later [12]. However, follow-up PET-CT in our patient after therapy yielded negative results (data not shown).

Patients with T3/T4 hypopharyngeal SCC usually require total laryngopharyngectomy. Because our patient initially had bulky stage IV disease, associated with frank thyroid cartilage invasion, total laryngopharyngectomy was applicable. However, the patient desired organ preservation and therefore chose chemoradiotherapy as an alternative to total laryngopharyngectomy [5]. As a result, the patient retained appreciable vocal cord function of speech and deglutition, and his prognosis was good at two years after primary radiotherapy without tumor recurrence. The return of vocal cord function after chemoradiotherapy is a useful prognostic factor, with a five-year overall survival rate in patients with recovered vocal cord function of 100%, compared with a two-year overall survival rate of patients with vocal cord fixation of less than 40% [13]. Our patient recovered vocal cord fixation, with no obvious signs of tumor recurrence more than two years after initial chemoradiotherapy. Therefore, chemoradiotherapy was appropriate for this patient.

## Conclusions

A patient developed a condition after chemoradiotherapy for hypopharyngeal cancer that required differential diagnosis between radionecrosis and tumor recurrence. Our findings emphasize the wide spectrum of radiographic presentation of radionecrosis.

## Abbreviations

CR: complete response; CT: computed tomography; SCC: squamous cell carcinoma.

## Consent

Written informed consent was obtained from the patient for publication of this case report and any accompanying images. A copy of the written consent is available for review by the Editor-in-Chief of this journal.

## Competing interests

The authors declare that they have no competing interests.

## Authors' contributions

All authors provided an equal intellectual contribution to this manuscript. All authors read and approved the final manuscript.
